# Quantitative evaluation of the efficacy of non-replicating vaccines for controlling African swine fever in domestic pigs: a systematic review and meta-analysis

**DOI:** 10.3389/fvets.2025.1614479

**Published:** 2025-09-25

**Authors:** Eurade Ntakiyisumba, Maryum Tanveer, Fabrice Hirwa, Gayeon Won

**Affiliations:** Bio-Safety Research Institute and College of Veterinary Medicine, Jeonbuk National University, Iksan, Republic of Korea

**Keywords:** African swine fever virus, inactivated, subunit, vaccine efficacy, meta-analysis

## Abstract

**Background:**

The African swine fever virus (ASFV), prevalent globally, causes high mortality and morbidity in domestic pigs. However, there is a lack of effective treatment or vaccines against ASFV infection despite the ongoing research in this field.

**Methods:**

In this systematic review and meta-analysis, we conducted a quantitative evaluation of the efficacy of non-replicating vaccines against ASFV. The vaccine efficacy (VE) was analyzed based on three key disease outcomes: mortality, fever, and clinical symptoms after infection.

**Results:**

The search of relevant electronic databases yielded 23 studies for inclusion in the review. Vaccination with subunit vaccines significantly reduced mortality risk in vaccinated pigs compared to that in controls (*p* = 0.02), with a relative risk (RR) of 0.90 (95% CI: 0.83–0.98), indicating a VE of 10% (95% CI: 2–17). However, subunit vaccines did not substantially reduce the risk of fever and other clinical symptoms in vaccinated pigs, with a RR of 0.97 (95% CI: 0.93–1.01) for both outcomes. Moreover, inactivated vaccines did not provide any protection against mortality (RR = 1.01, 95% CI: 0.95–1.06) or other clinical signs (RR = 1.00, 95% CI: 1.00–1.00). No significant between-study heterogeneity was detected, indicating consistent findings across different vaccination trials. Thus, currently available non-replicating vaccines fail to deliver the protection required for field applications.

**Conclusion:**

Currently, subunit vaccines are more likely to serve as long-term options for vaccine development strategies. Further research is essential to deepen our understanding of the roles and significance of humoral and cellular immune responses against ASFV, and to identify critical viral antigens that can induce effective protective immunity.

## 1 Introduction

African swine fever (ASF) is a highly contagious and deadly disease that affects domestic and wild members of the Suidae family. The disease was first identified in Africa in the 1920s and subsequently spread to Europe, Asia, and the Americas in the following decades (1). It is caused by the African swine fever virus (ASFV), a large enveloped DNA virus of the *Asfarviridae* family, which is characterized by a complex icosahedral structure and a genome that encodes over 150 proteins (2). The virus displays considerable genotypic diversity, with 24 genotypes identified based on the nucleotide sequence diversity of the B646L gene, which encodes the major capsid protein p72 (3). ASFV exhibits pronounced phenotypic variability, driven in large part by its immune-evasion mechanisms that modulate innate and adaptive host defenses (4). ASFV spreads through direct contact with infected pigs, contaminated pork products, and biological vectors such as soft ticks of the *Ornithodoros* genus, which can host the virus for long durations (5, 6). Its high environmental stability further facilitates disease transmission. Since the disease was first identified in Africa, it has spread globally and has had a significant economic impact on the swine industry (1). Acute clinical forms of ASF manifest as high fever, hemorrhage, respiratory distress, and cause up to 100% mortality, whereas subacute and chronic forms present milder symptoms and have lower mortality rates depending on the viral strain (7, 8). Owing to the absence of effective vaccines or treatments, ASFV continues to pose a significant threat to swine populations worldwide, thereby emphasizing the need for ongoing research and improved control measures to mitigate its spread and impact (6).

In the pursuit of developing an effective ASFV vaccine, live attenuated vaccines (LAVs) have frequently demonstrated the most robust protection as discussed in our previous study (9). However, despite their high efficacy, the deployment of LAVs is hampered by significant safety concerns, including post-vaccination fever, clinical reactions, and lingering risks of reversion to virulence or chronic infection (10, 11). Unlike LAVs, subunit and inactivated vaccines (hereafter referred to as non-replicating vaccines) do not use live pathogens, thereby eliminating the risk of reversion to a virulent form. A notable benefit of non-replicating vaccines, particularly subunit vaccines, is their ability to achieve high expression levels, while maintaining low production costs. Additionally, these vaccines enable the differentiation of infected from vaccinated animals (DIVA) by excluding specific antigens or encoding immunogens that can serve as vaccine markers, allowing companion diagnostic tests to distinguish infected from vaccinated animals (1, 12). Over the past few decades, non-replicating vaccines have become essential for managing infectious diseases in pigs and have been highly effective in promoting animal health, lowering mortality rates, and boosting productivity in swine populations (13–15). Notably, recombinant subunit vaccines for diseases such as classical swine fever (16–18), foot-and-mouth disease (19), and porcine circovirus type 2 (20–23) have been successfully developed and approved by regulatory bodies. An inactivated vaccine targeting porcine parvovirus, a major contributor to reproductive failure and economic losses in pigs, has also undergone field testing (13, 24). These advancements highlight the critical role of non-replicating vaccines as indispensable tools for disease control and productivity improvement in modern pig farming. Given the safety concerns associated with ASF LAVs, non-replicating vaccines have been explored as alternative approaches that offer several advantages, particularly in terms of safety and stability. While subunit and inactivated vaccines offer enhanced safety, stability, and DIVA compatibility, they come with notable drawbacks. These vaccine platforms typically induce weaker immune responses compared to LAVs, especially in terms of inducing cellular immunity such as CD8^+^ T-cell responses (25, 26). Moreover, non-replicating vaccines often require adjuvants and multiple booster doses to achieve and maintain protection (25). Given these trade-offs, it is imperative to comprehensively assess the feasibility of non-replicating vaccines as safer means to combat ASFV in endemic and at-risk regions.

Subunit vaccines can be formulated to focus the immune response on specific components of the pathogen, such as its surface proteins, to enhance the specificity of the immune response and reduce the risk of unwanted side effects (10, 27, 28). Subunit vaccines consist of purified antigens specifically derived from pathogenic microorganisms that stimulate the host’s immune system to produce targeted antibodies (29). These vaccines contain proteins, peptides, or polysaccharides with immunogenic epitopes that trigger an immune response (12, 27, 29). Despite the genetic diversity of ASFV, several of its proteins, including p12, CD2v, p72, pp220, p54, p30, and pp62, involved in different stages of viral attachment and internalization have been reported to be immunogenic (7, 12, 30). For instance, antibodies targeting p12, p72, or p54 block viral adsorption, whereas antibodies against p30 inhibit ASFV internalization (27, 31, 32). These discoveries have intensified the focus on these proteins for developing recombinant protein-based, DNA-based, and viral-vectored vaccines against ASFV. Inactivated vaccines rely on physical or chemical inactivation of the virus and are often combined with specific adjuvants to improve immune responses (33–35).

Several reviews have examined the research on non-replicating vaccines against ASF (1, 4, 7, 10, 12, 27–29, 31, 32, 36–40) and have provided valuable insights into the strategies used for formulation and administration, the immunogenicity, and potential protective effectiveness of these vaccines in domestic pig populations. Despite these thorough reviews, there is a significant lack of quantitative assessments of the efficacy of non-replicating vaccines against ASF in domestic pigs. This lack limits our complete understanding of the effectiveness of these vaccines in ASF control, and underscores the need for additional research to address this knowledge gap. Meta-analysis serves as a valuable tool in veterinary science for evaluating the efficacy and safety of treatments aimed at enhancing animal health, productivity, and reproduction (41, 42). It combines data from multiple independent studies on a specific topic to produce stronger and more statistically powerful estimates than those obtained from individual studies (43–45). This approach offers more reliable insights into a research topic and helps identify sources of variation among the study results (44). Thus, to address the gaps in the current knowledge on the efficacy of non-replicating ASF vaccines, we conducted a systematic review and meta-analysis (SRMA) of controlled ASF vaccine trials to evaluate the effectiveness of inactivated and subunit vaccines against virulent ASFV strains in domestic pigs to offer critical insights into their potential role in ASF control.

## 2 Materials and methods

### 2.1 Research question and search strategy

This review was conducted in accordance with the Preferred Reporting Items for Systematic Reviews and Meta-Analyses (PRISMA) guidelines (46). The PRISMA guideline checklist for this study is provided in Supplementary material 1. The review question was structured using the “PICO” (population, intervention, comparator, and outcome) framework. The “population” included domestic pigs; “intervention” involved pigs vaccinated with inactivated or subunit vaccines against ASFV infection; the “comparator” group included unvaccinated pigs or those administered a placebo; and key disease “outcomes” included mortality rates, fever, and clinical signs associated with ASF in both vaccinated and control groups. A comprehensive literature search was performed across the international databases MEDLINE (via PubMed), Web of Science, and Scopus, along with several Korean databases including RISS, KISS, and ScienceOn, to identify relevant studies published until May 28, 2024. The following search terms were used: (ASF OR African swine fever OR ASFV OR African swine fever virus) AND (immun* OR vaccin* OR interven* OR treatment OR efficacy OR safety OR effect OR protect* OR mitigat* OR control OR antibody OR prevent* OR subunit OR DNA OR recombinant OR vector OR candidate OR antigen OR inactivated OR killed) AND (swine OR pigs OR piglets OR gilts OR sows OR weaner OR feeders OR finishers).

### 2.2 Inclusion and exclusion criteria

Primary studies investigating the efficacy of inactivated and/or subunit vaccines against ASF in domestic pigs of any breed in an experimental challenge model were included in the review. Studies that evaluated vaccine efficacy (VE) against virulent ASFV strains were considered eligible. The VE should have been assessed based on at least one clinical disease outcome such as mortality, fever, or ASF-related symptoms. No language restrictions were imposed in the inclusivity criteria. Studies involving animals other than domestic pigs, those using tick bites as the challenge method, review articles, and *in vitro* studies were excluded from the review. In this study, the VE was defined as the ability to confer protection against disease and prevent the clinical manifestations of ASFV infection. Therefore, studies focusing on humoral and/or cellular immune responses rather than clinical outcomes were excluded, as immune responses are regarded proxy indicators of protection, and the presence of antibodies does not consistently correlate with protection against ASFV infection (47, 48). Furthermore, experimental studies without control groups or full-text access were also excluded. After screening the titles and abstracts, the relevant articles were reviewed completely to confirm their eligibility.

### 2.3 Data extraction

Three independent reviewers (EN, MT, and FH) extracted data from the eligible studies, and any discrepancies were resolved through discussion and consensus. The extracted information included author names; publication year; country; target antigen/protein; source strain; vaccine type; vaccine dose; vaccination route; name of the adjuvant; challenge strain; challenge dose; number of animals in each experimental group; and disease outcomes such as mortality, fever, and other clinical signs. All outcomes were considered dichotomous variables, and the number of animals that experienced each outcome was recorded. Apart from fever, all reported ASF symptoms (e.g., lethargy, anorexia, recumbence, cyanosis, skin hemorrhages, joint swelling, labored breathing, coughing, ocular discharge, and digestive disorders) were collectively grouped and analyzed as the “clinical signs” outcome. Animals exhibiting at least one of the above symptoms after ASFV challenge were classified as diseased, as long as the study author attributed the symptom to ASFV infection. This approach was deemed appropriate due to the broad range of symptoms associated with ASFV infection in domestic pigs, making it unlikely for an animal to display all symptoms simultaneously. On the other hand, animals explicitly reported by the author as not developing any ASFV-attributable symptom were considered fully protected.

### 2.4 Risk-of-bias assessment

The internal and external validity of the included studies was assessed by three independent reviewers (EN, MT, and FH) against the Animal Research: Reporting *in vivo* Experiments 2.0 (ARRIVE 2.0) guidelines checklist (49). Eighteen RoB items were assessed and each was rated as either low, unclear, or high RoB. Discrepancies were resolved through discussions until a consensus was reached.

### 2.5 Statistical analyses of the data

The VE was assessed by examining three main disease outcomes: mortality, fever, and clinical signs after infection. As mentioned previously, all ASF clinical signs reported in the primary studies (excluding fever) were collectively grouped and analyzed under the “clinical signs” outcome. The meta-analysis was undertaken using R statistical software, version 4.1.2, employing the “meta,” “metafor,” and “dmetar” packages (50–53). The effect of vaccination was estimated using risk ratios (RR) and their corresponding 95% confidence intervals (CI). The VE was calculated as the percentage of cases preventable through vaccination, using the formula (1 - RR) * 100 (54). The 95% CIs for VE were determined using the substitution method (55). A random-effects model was used to combine effect sizes, accounting for the expected heterogeneity across studies. The Mantel–Haenszel method was employed to assign weights to each study during data pooling (56). The between-study variance of true effect sizes (τ^2^), was estimated using the Paule-Mandel estimator (57), whereas the Knapp-Hartung adjustment was applied to stabilize variance and calculate CIs around the pooled effect estimates (58). The presence of between-study heterogeneity was evaluated using Cochran’s statistic and the *I^2^* test (59). The *Q*-statistic was used to test the null hypothesis that all studies in the analysis shared a common effect size, whereas the *I^2^* statistic indicated the proportion of observed variance due to true effects rather than sampling error. Heterogeneity was considered significantly high if *I^2^* was > 50% and the *p*-value was < 0.05. A subgroup analysis was conducted to compare effect sizes across groups and to identify moderators associated with variability in effect sizes. Publication bias was assessed by visual inspection of the funnel plot symmetry and quantitatively using Egger’s regression test (60). When publication bias was confirmed, the Duval and Tweedie trim-and-fill method was applied to estimate the unbiased effect by imputing missing studies into the funnel plot (61). The trim-and-fill method is a nonparametric, funnel-plot-based data-augmentation technique that addresses potential publication bias by evaluating asymmetry in a funnel plot. It iteratively removes the most extreme small-study effects causing the asymmetry and then fills by imputing mirror-image studies to restore symmetry (62). This strategy enables recalculation of a more balanced pooled effect estimate.

## 3 Results

### 3.1 Search results

**Figure 1:**
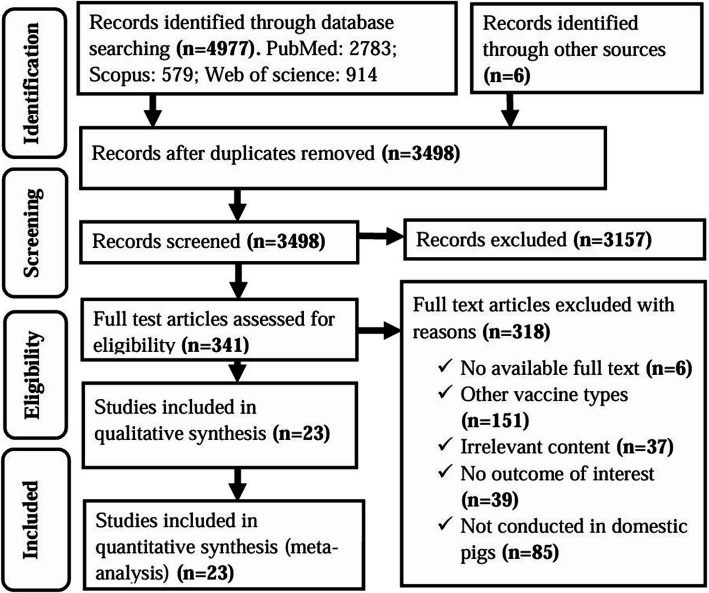
PRISMA flow diagram of the selection of studies for use in a systematic review and meta-analysis of the efficacy of non-replicating vaccines against ASF in domestic pigs.

A comprehensive search initially identified 4,983 studies from electronic databases and other sources. After removal of duplicates, 3,498 studies were subjected to title and abstract screening. Of these, 3,157 were deemed irrelevant and excluded from the review. Thus, 341 studies remained for full-text review, of which a further 318 were excluded for reasons such as the use of vaccines other than inactivated or subunit, lack of relevant clinical outcomes, unrelated data, missing full text, or study focus outside domestic pigs. Finally, 23 studies met the inclusion criteria and were incorporated into both qualitative and quantitative analyses; 19 studies were on subunit vaccines and 4 on inactivated vaccines (Figure 1). According to Rodrigues et al. (2015), vaccine designs such as virus-like particles (VLPs), DNA vaccines, vectored vaccines, and vectored VLPs are often classified as subunit vaccines because they deliver only specific antigens of the pathogen, either as proteins or genetic material (63). Consistent with this classification, the subunit vaccines evaluated in this study include protein-, DNA-, and viral vector-based vaccines.

### 3.2 Study characteristics

**Figure 2:**
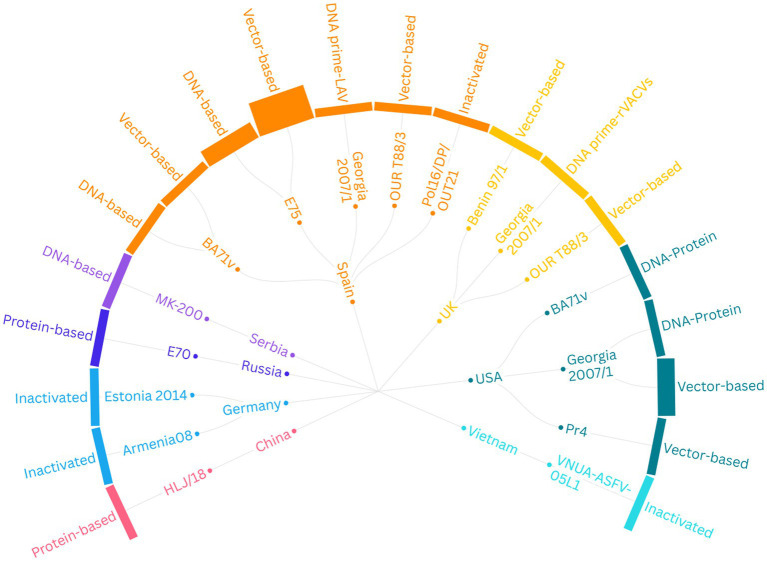
Summary of the experimental designs of 23 studies evaluating the efficacy of non-replicating vaccines against ASFV in domestic pigs. The graph’s structure moves from the outer layer to the center, displaying (1) the type of vaccine, (2) the viral strain from which the antigen was derived, and (3) the country where the study was conducted. LAV, Live Attenuated Vaccine (BA71ΔCD2); rVACVs, Recombinant Vaccinia Viruses; USA, United States of America; UK, United Kingdom.

After a comprehensive evaluation of the identified records, 23 studies met the inclusion criteria of the SRMA. Regarding subunit vaccines, 10 studies were conducted in Spain, four in the United States (USA), two in the United Kingdom (UK), and one each in China, Serbia, and Russia. In terms of vaccine type, 10 studies investigated viral vectored vaccines (64–73), two focused on protein-based vaccines (74, 75), four focused on DNA-based vaccines (47, 76–78), and the remaining three explored combination or heterologous prime-boost vaccination strategies (Figure 2). For instance, one study examined a combined DNA-protein vaccine (79), another primed pigs with a DNA-based vaccine and provided a booster of recombinant vaccinia viruses (80), whereas a third study involved priming twice with plasmid DNA followed by a boost with BA71∆CD2 (81), a recombinant live attenuated vaccine. For vaccine delivery, 13 studies employed intramuscular immunization, two used the subcutaneous route, and four studies utilized a combination of both routes. Detailed information regarding the experimental design of studies investigating the efficacy of subunit vaccines against ASFV in domestic pigs is provided in Table 1. Regarding the inactivated vaccines, two studies were conducted in Germany (33, 34), one in Spain (26), and one in Vietnam (82). In terms of the inactivation method, three studies utilized chemical inactivation with binary ethylenimine (BEI) (26, 33, 82), whereas one study employed gamma irradiation (34). Regarding the vaccination route, three studies employed intramuscular immunization, and one used both intramuscular and intradermal routes. Table 2 depicts the main characteristics of the studies assessing the efficacy of inactivated vaccines against ASF in domestic pigs.

**Table 1:** Characteristics of 19 studies included in the systematic review and meta-analysis of the efficacy of subunit vaccines against African swine fever in domestic pigs.

Author/Year	Vaccine type	Target antigen	Source strain	Vaccine dose	Vaccine route	Adjuvant	Challenge strain (Dose)
Ivanov et al. (74)	Protein-based	p158, p327, p14, p10, p11.5	E70	2.5 mL	SC	Freund’s adjuvant	E70 (10^3^ HAU_50_)
	Protein-based	S273R, p22	E70	2.5 ml	SC	Freund’s adjuvant	E70 (10^3^ HAU_50_)
	Protein-based	p30 (CP204L), p72 (B646L)	E70	2.5 ml	SC	Freund’s adjuvant	E70 (10^3^ HAU_50_)
	Protein-based	p54 (E183L)	E70	2.5 ml	SC	Freund’s adjuvant	E70 (10^3^ HAU_50_)
Hua et al. (75)	Protein-based	CD2v (EP402R)	HLJ/18	1 mL (50 μg)	IM	MONOTA VG 61 (SEPPIC)	HLJ/18 (300 HAD_50_)
	Protein-based	CD2v (EP402R), p30 (CP204L)	HLJ/18	50 μg per antigen	IM	MONOTA VG 61 (SEPPIC)	HLJ/18 (300 HAD_50_)
	Protein-based	CD2v (EP402R), p30 (CP204L), K205R	HLJ/18	50 μg per antigen	IM	MONOTA VG 61 (SEPPIC)	HLJ/18 (300 HAD_50_)
Imatdinov et al. (78)	DNA-based	CD2v (EP402R), p30 (CP204L), p54 (E183L)	MK-200	1.5 ml	IM	_^g^	M-78 (10^3^ HAU_50_)
Argilaguet et al. (47)	DNA-based	p54 (E183L), p30 (CP204L)	E75	600 μg	IM & SC	_	E75 (10^4^ HAU_50_)
	DNA-based	SLA-II/p54 (E183L)/p30 (CP204L)	E75	600 μg	IM & SC	APCH1	E75 (10^4^ HAU_50_)
Argilaguet et al. (76)	DNA-based	p54/p30	E75	600 μg	IM & SC	_	E75 (10^4^ HAU_50_)
	DNA-based	sHA/p54/p30 fusion	E75	600 μg	IM & SC	sHA	E75 (10^4^ HAU_50_)
	DNA-based	Ubiquitin/sHA/p54/p30/ fusion	E75	600 μg	IM & SC	sHA	E75 (10^4^ HAU_50_)
Lacasta et al. (77)	DNA-based	80 ORFs fragments fused with Ubiquitin	BA71v	600 μg	IM & SC	_	E75 (10^4^ HAU_50_)
Jancovich et al. (80)^a^	DNA prime-rVACVs	A pool of 47 antigens	Georgia 2007/1	Prime: 10 μg Boost: 10^8^ PFU	SC	CpG oligonucleotide	Georgia 2007/1 (10^4^ HAU_50_)
Sunwoo et al. (79)	DNA-Protein	CD2v, p72, p32, p15, p35, p54	Georgia 2007/1, BA71v	100 μg pcDNA + 100 μg proteins	IM	ISA25 adjuvant (SEPPIC)	Arm07 (360 HAU)
	DNA-Protein	CD2v, p72, p32, p15, p35, p54, p17	Georgia 2007/1, BA71v	100 μg pcDNA + 100 μg proteins	IM	ISA25 adjuvant (SEPPIC)	Arm07 (360 HAU)
Bosch-Camos et al. (81)^b^	DNA prime-LAV	Ub/M448R/MGF505-7R	Georgia 2007/1	Prime: 0.6 mg Boost: 10^3^ PFU	IM	_	Georgia 2007/1 (10^3^ GEC)
	DNA prime-LAV	B475L, B602L, CP2475L, D339L, DP238L, EP424R, H339R, I226R, I243L, I73R, I9R, K145R, M448R, MGF505-1R, MGF505-3R	Georgia 2007/1	Prime: 0.6 mg Boost: 10^3^ PFU	IM	_	Georgia 2007/1 (10^3^ GEC)
Argilaguet et al. (64)^c^	Vector-based	sHA/p54/p30 fusion	E75	10^7^ PFU	IM & SC	_	E75 (10^2^ HAU_50_)
Lokhandwala et al. (65)^d^	Vector-based	A151R, B119 L, B602 L, EP402RΔPRR, B438L, K205R-A104R, pp62, B646L	Georgia 2007/1	Prime: 8 × 10^10^ IFU Boost: 8×10^11^ IFU	IM	BioMize (VaxLiant)	Georgia 2007/1 (10^4^ TCID_50_)
	Vector-based	p32, p54, pp62, p72, p37, p150-I, p150-II.	Georgia 2007/1	Prime: 7 × 10^10^ IFU Boost: 7×10^11^ IFU	IM	ZTS-01 (Zoetis)	Georgia 2007/1 (10^3^ TCID_50_)
	Vector-based	p32, p54, pp62, p72, p37, p150-I, p150-II.	Georgia 2007/1	Prime: 7 × 10^10^ IFU Boost: 7×10^11^ IFU	IM	BioMize	Georgia 2007/1 (10^3^ TCID_50_)
Netherton et al. (66)^d^	Vector-based	A151R, p72, C129R, p30, p54, E146L, I215L, I73R, L8L, M448R, MGF110-4 L, MGF110-5 L	OUR T88/3	5 × 10^9^ IFU	IM	_	OUR T88/1 (10^4^ HAD_50_)
	Vector-based	A151R, p72, C129R, p30, p54, E146L, I215L, I73R, L8L, M448R, MGF110-4 L, MGF110-5 L	OUR T88/3	Prime: 5 × 10^9^ IFU Boost: 7.5 × 10^9^ PFU	IM	_	OUR T88/1 (10^4^ HAD_50_)
Zajac et al. (67)^d^	Vector-based	pcDNA3 plasmids encoding 42 antigens	Georgia 2007/1	4.2 × 10^11^ IFU	IM	_	Georgia 2007/1 (10^2^ TCID_50_)
	Vector-based	pcDNA3 plasmids encoding 42 antigens	Georgia 2007/1	4.2 × 10^11^ IFU	IM	Montanide ISA-201™	Georgia 2007/1 (10^2^ TCID_50_)
	Vector-based	pcDNA3 plasmids encoding 42 antigens	Georgia 2007/1	4.2 × 10^11^ IFU	IM	BioMize	Georgia 2007/1 (10^2^ TCID_50_)
Goatley et al. (68)^e^	Vector-based	B602L, B646L, CP204L, E183L, E199L, EP153R, F317L, MGF505-5R	OUR T88/3, Benin 97/1	Prime: 5 × 10^9^ IFU Boost: 7.5 × 10^7^ PFU	IM	_	OUR T88/1 (10^4^ HAD)
	Vector-based	B602L, EP153R, EP364R, F317L, I329L, MGF360-11 L, MGF505-4R, MGF505-5R	OUR T88/3, Benin 97/1	Prime: 5 × 10^9^ IFU Boost: 7.5 × 10^7^ PFU	IM	_	OUR T88/1 (10^4^ HAD)
Carrascosa et al. (69)^f^	Vector-based	p12	BA71v	0.5 mg	IM	Freund’s adjuvant	E70 (10^3^ TCID_50_)
Ruiz-Gonzalvo et al. (70)^f^	Vector-based	CD2v (EP402R)	E75	10^7^ HAU_50_	IM	Freund’s adjuvant	E75 (4×10^2^ TCID_50_)
	Vector-based	CD2v (EP402R)	E75	5×10^6^ HAU_50_	IM	Freund’s adjuvant	E75 (4×10^2^ TCID_50_)
Gomez-Puertas et al. (71)^f^	Vector-based	p30 (CP204L)	E75	100 μg	IM	Freund’s adjuvant	E75 (5×10^2^ TCID_50_)
	Vector-based	p54 (E183L)	E75	100 μg	IM	Freund’s adjuvant	E75 (5×10^2^ TCID_50_)
	Vector-based	p54 (E183L), p30 (CP204L)	E75	100 μg	IM	Freund’s adjuvant	E75 (5×10^2^ TCID_50_)
Barderas et al. (72)^f^	Vector-based	p54/p30 chimera	E75	100 μg	IM	Freund’s adjuvant	E75 (5×10^2^ TCID_50_)
Neilan et al. (73)^f^	Vector-based	p54 (E183L), p30 (CP204L), p72 (B646L), p22	Pr4	200 μg	IM	Freund’s adjuvant	Pr4 (10^4^ TCID_50_)

ORFs, Open reading frames; IFU, Infectious units; PFU, Plaque forming units; HAU, Hemadsorbing units; HAD, Hemadsorption; TCID, Tissue culture infectious doses; GEC, Gene equivalent copies; SC, Subcutaneous; IM, Intramuscular; APCH1, MHC Class II DR molecule; sHA, Extracellular domain of the ASFV Hemagglutinin. ^a^Primed with DNA-based vaccine and booted with recombinant vaccinia viruses (rVACVs). ^b^Primed with DNA-based vaccine and boosted with a live attenuated vaccine (BA71∆CD2). ^c^Antigens were delivered in a BacMam vector. ^d^Antigens were delivered in Adenovirus vector. ^e^Primed with genes vectored by Adenovirus and boosted with Modified vaccinia Ankara (MVA). ^f^Antigens were delivered in Baculovirus vector. ^g^No adjuvant was used.

**Table 2:** Characteristics of 4 studies included in the meta-analysis of the efficacy of inactivated vaccines against African Swine Fever (ASF) in domestic pigs.

Author/Year	Country	ASFV strain	Inactivation method	Vaccine dose	Adjuvant	Challenge strain (Dose)
Blome et al. (33)	Germany	Armenia08	Chemical	5 ml	Polygen™, Emulsigen®-D	Armenia08 (10^9^ TCID_50_)
Cadenas-Fernández et al. (26)	Spain	Pol16/DP/OUT21	Chemical	6×10^9^ HAD_50_	MF59®, Silica oil, mGNE, Montanide™ ISA201 VG,	Pol16/DP/OUT21 (10 HAD_50_)
Pikalo et al. (34)	Germany	Estonia 2014	Irradiation	2 mL	Polygen™, Montanide™ ISA 201 VG	Armenia08 (10^6^ HAU)
Luong et al. (82)	Vietnam	VNUA-ASFV-05 L1	Chemical	5×10^7^ HAD_50_	Montanide™ ISA 201 VG	VNUA-ASFV-05 L1 (7×10^2^ HAD_50_)

### 3.3 Quality assessment

**Figure 3:**
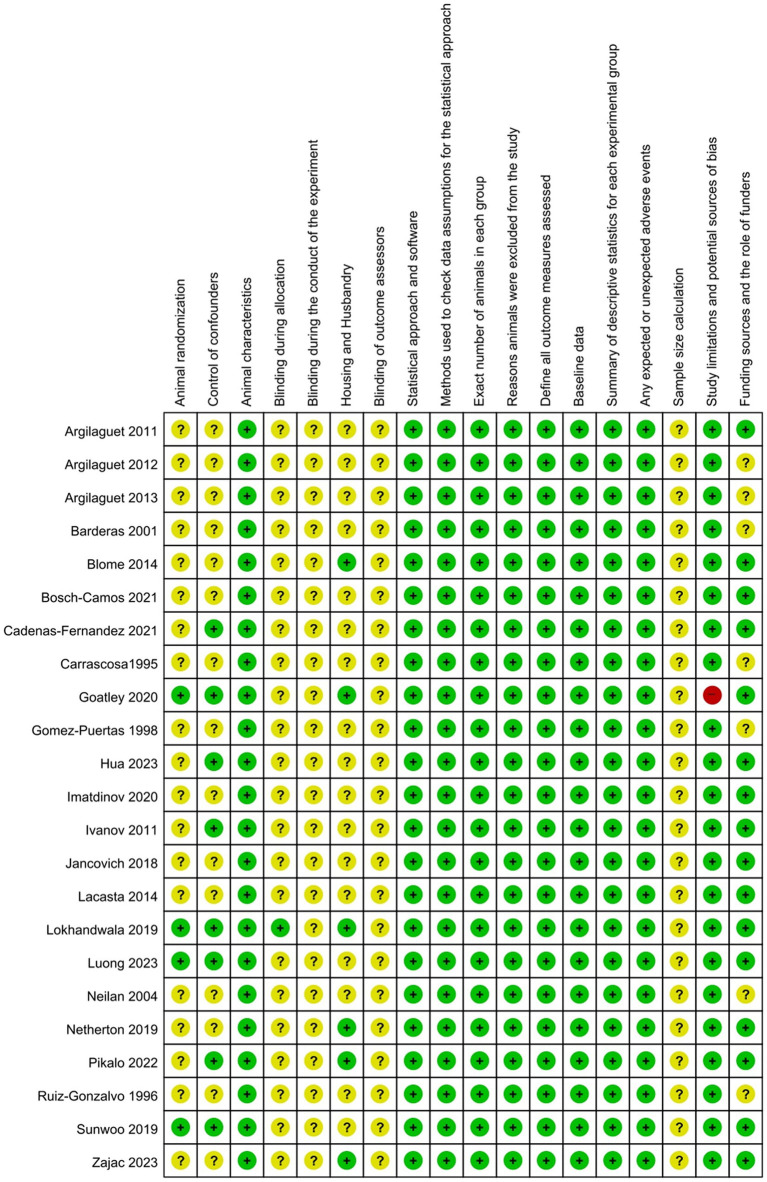
Risk-of-bias assessment of the eligible studies based on the ARRIVE guidelines checklist. Green color denotes a low risk of bias, red denotes a high risk of bias, and yellow indicates an unclear risk of bias.

All 23 studies included in the SRMA were evaluated for potential RoB using the ARRIVE 2.0 guidelines checklist, which assesses 18 specific items (Figure 3). Notably, no study demonstrated a consistently low RoB across all evaluated domains. All studies (100%) did not provide details on sample size determination or personnel blinding during experiments and outcome assessments, and they were rated with an unclear RoB for these items. In one study (68), some vaccinated animals showed ASF-related clinical signs after challenge and were treated with the nonsteroidal anti-inflammatory and antipyretic drug flunixin meglumine. This study was rated as high RoB for the “Study limitations and potential sources of bias” category, as the protective effect observed in the animals was likely influenced by the treatment rather than solely by the vaccine. Seven studies (30.4%) did not disclose their funding sources or the role of the funders, leading to an unclear RoB, whereas the remaining studies (69.6%) were rated as low RoB in this category. Regarding animal randomization, four studies (17.4%) described their randomization strategies and eight studies (34.8%) used antibody-negative pigs or randomly assigned pigs in experimental groups, earning these studies a low RoB rating for randomization or control of cofounders. In summary, most studies were judged to have either a low or unclear RoB across the 18 items, based on the ARRIVE 2.0 guidelines.

### 3.4 Meta-analysis of subunit vaccines

#### 3.4.1 Mortality outcome

**Figure 4:**
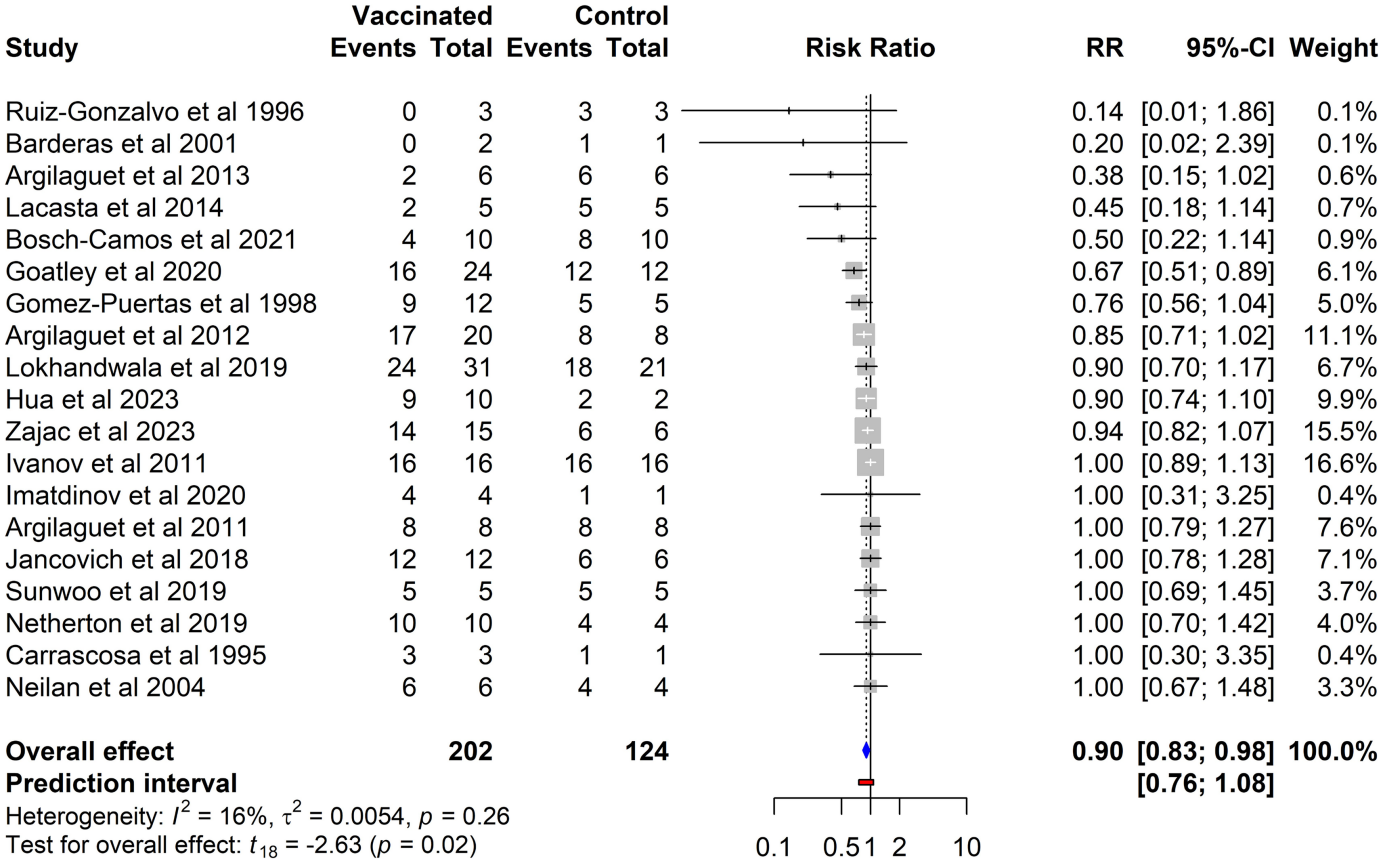
Forest plot of the efficacy of subunit vaccines against ASF in domestic pigs based on mortality outcome. The pooled effect estimate is shown as the RR with its corresponding 95% confidence interval, as calculated using a random-effects model.

**Figure 5:**
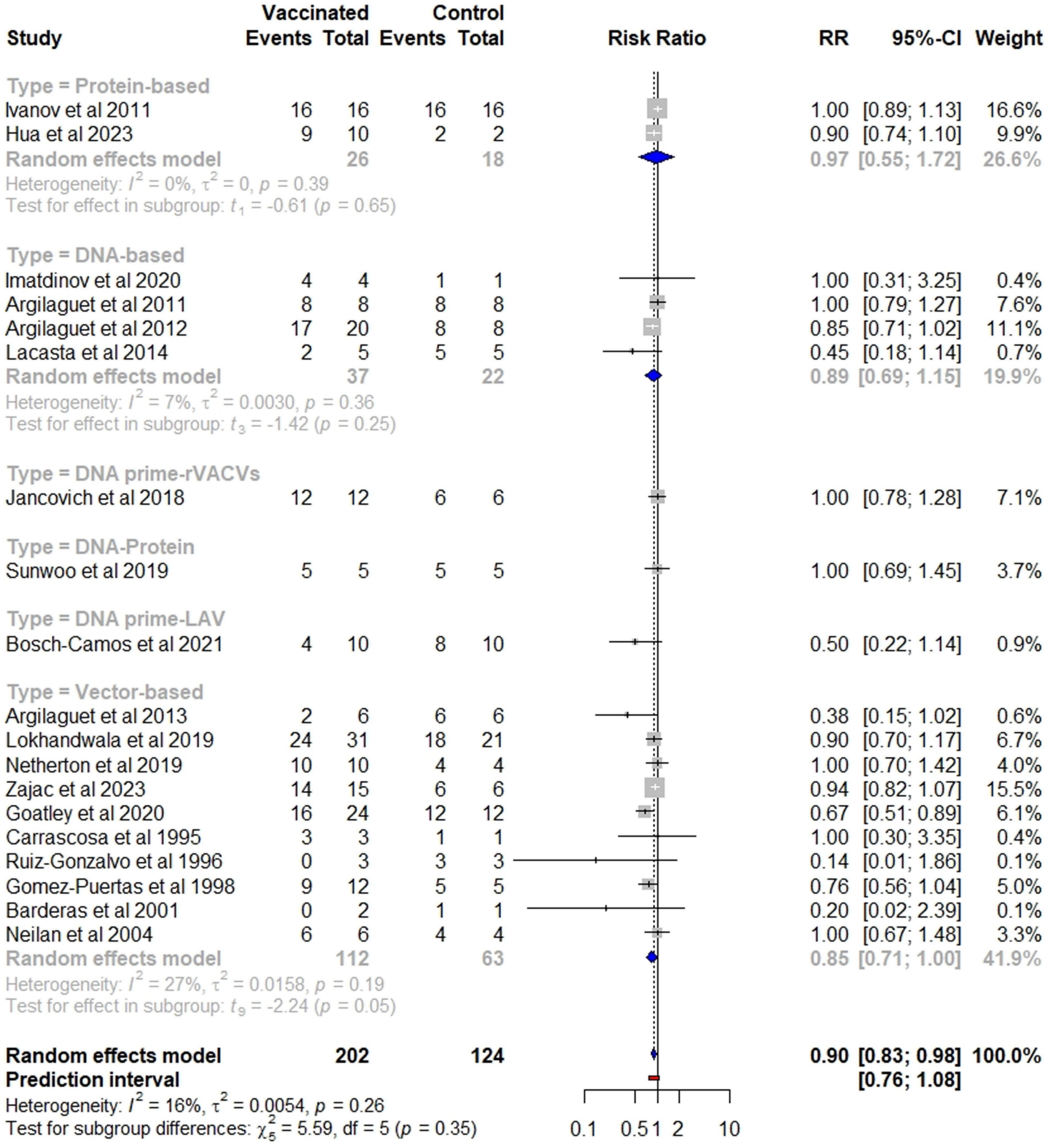
Subgroup analysis of the effectiveness of subunit vaccines in preventing mortality, categorized by vaccine type.

Overall, vaccinated pigs had a significantly lower risk of mortality compared to the controls (*p* = 0.02), with a pooled RR of 0.90 (95% CI: 0.83–0.98). This effect size corresponds to a VE of 10% (95% CI: 2–17) (Figure 4). Specifically, viral-vectored vaccines exhibited the highest efficacy (RR = 0.85, 95% CI: 0.71–1), followed by DNA-based (RR = 0.89, 95% CI: 0.69–1.15), and protein-based (RR = 0.97, 95% CI: 0.55–1.72) vaccines, although the differences between these groups were not statistically significant (Figure 5). Furthermore, no significant heterogeneity was found (*I^2^* = 16, *p* = 0.26), indicating consistent findings across studies.

#### 3.4.2 Fever and clinical signs outcomes

**Figure 6:**
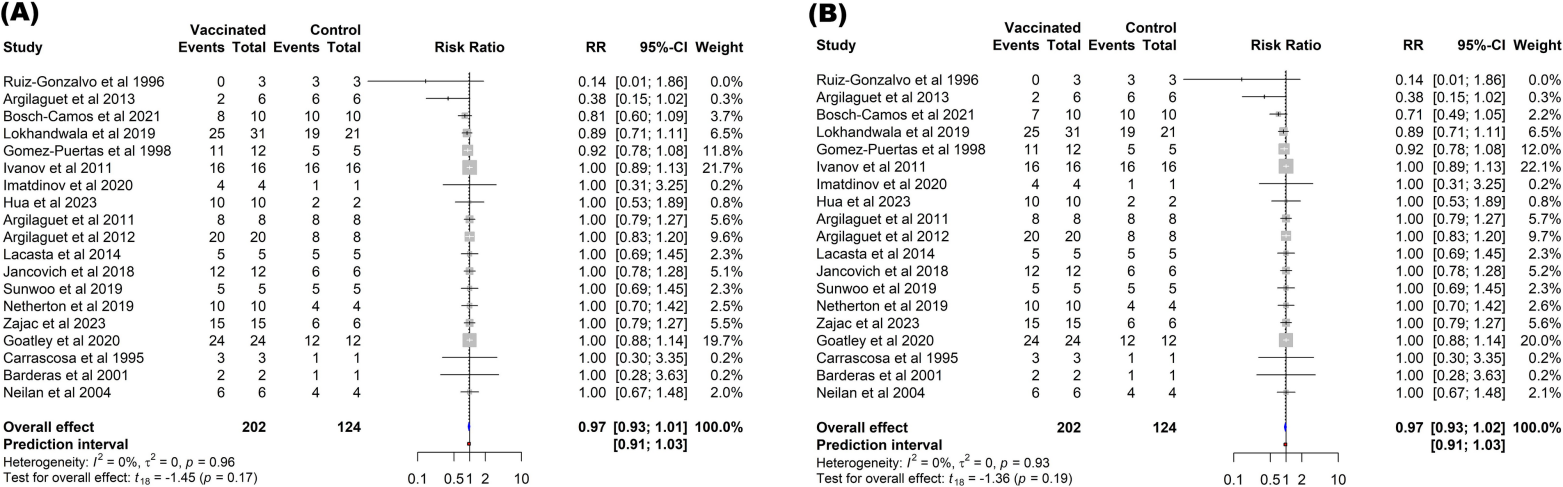
Forest plot of the efficacy of subunit vaccines against ASF in domestic pigs based on fever **(A)**, and clinical signs **(B)** outcomes. The pooled effect estimate is shown as the RR with its corresponding 95% confidence interval, as calculated using a random-effects model.

In contrast to the mortality outcome, the subunit vaccines did not significantly reduce the risk of fever (*p* = 0.17) or other ASF-associated clinical symptoms (*p* = 0.19) in vaccinated pigs compared to those in controls. The overall protection against fever among vaccinated pigs (RR = 0.97, 95% CI: 0.93–1.01) was comparable to the protection against other clinical signs (RR = 0.97, 95% CI: 0.93–1.02). These effect sizes corresponded to a VE of 3% (Figure 6).

### 3.5 Meta-analysis of inactivated vaccines

**Figure 7:**
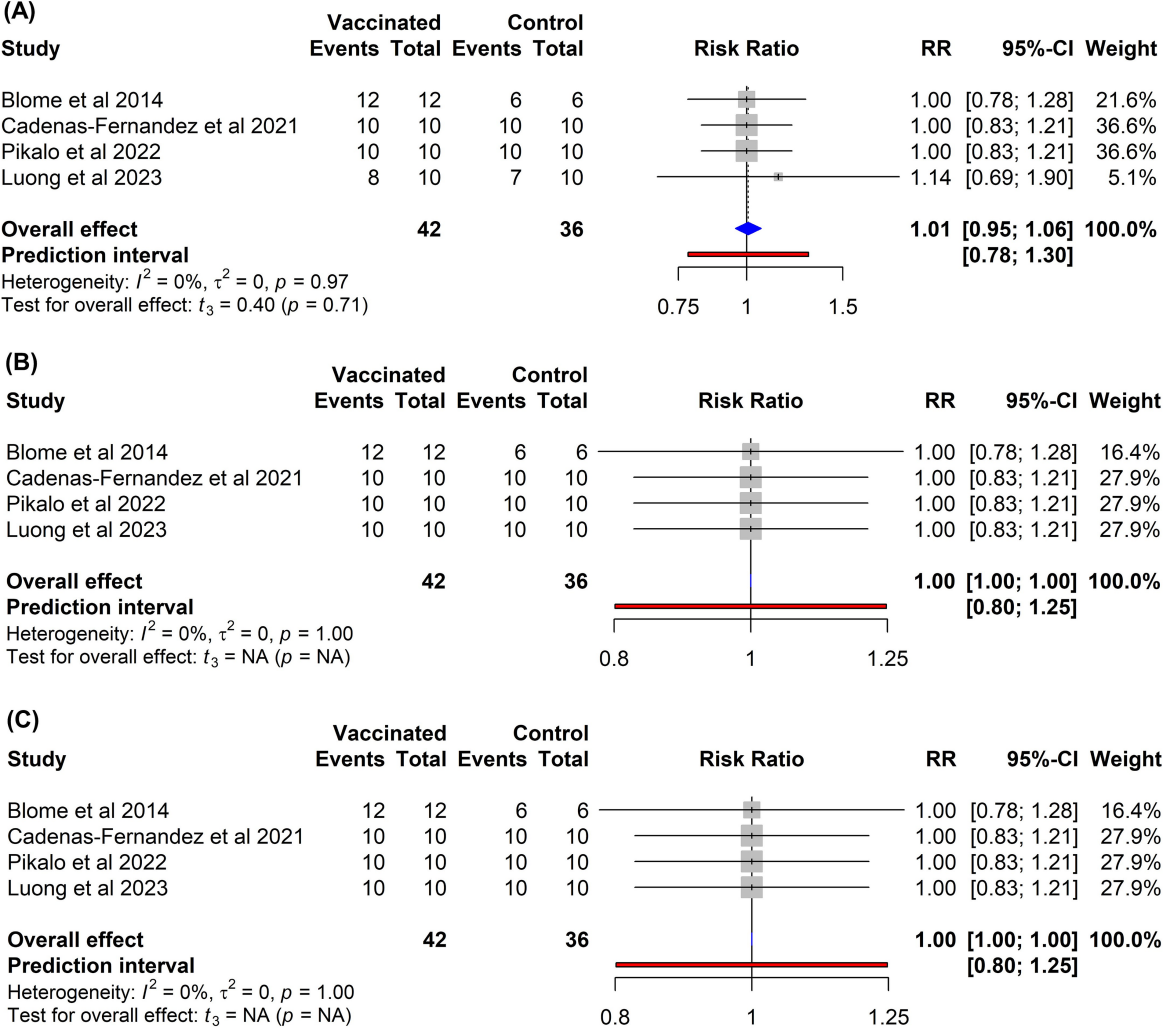
Forest plot of the efficacy of inactivated vaccines against ASF in domestic pigs based on mortality **(A)**, clinical signs **(B)**, and fever **(C)** outcomes. The pooled effect estimate is shown as the RR with its corresponding 95% confidence interval, as calculated using a random-effects model.

In contrast to the subunit vaccines, immunization of pigs with inactivated vaccines failed to confer protection (Figure 7). The risk of mortality among vaccinated pigs (RR = 1.01, 95% CI: 0.95–1.06) was comparable to that observed in non-vaccinated controls (*p* = 0.71). Similarly, currently developed inactivated vaccines did not provide any protection against fever (RR = 1.00, 95% CI: 1.00–1.00) or other clinical symptoms associated with ASF (RR = 1.00, 95% CI: 1.00–1.00) when compared to control groups.

### 3.6 Publication bias

**Figure 8:**
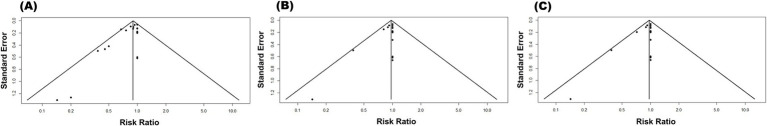
Funnel plot for publication bias on the efficacy of subunit vaccines in preventing ASF in domestic pigs considering the mortality **(A)**, clinical signs **(B)**, and fever **(C)** outcomes.

To evaluate the likelihood of publication bias, funnel plots were constructed with the effect size on the x-axis and standard error on the y-axis (Figure 8). Visual examination of these plots revealed an asymmetric distribution of studies for all outcomes, suggesting publication bias. Given the subjective nature of the funnel plot interpretation, Egger’s regression test was performed to further investigate the significance of funnel plot asymmetry. Egger’s test yielded statistically significant results for mortality (*t* = −3.015, *p* = 0.007), confirming the presence of publication bias for this outcome. To assess the influence of publication bias on the pooled effect size, the trim-and-fill method was used to generate a corrected estimate that accounted for potentially missing studies. The analysis identified three studies that were likely missing due to publication bias. Following the application of the trim-and-fill method, the adjusted RR was 0.89 (95% CI: 0.80–1.00). This adjusted effect size closely aligns with the initial pooled estimate (RR = 0.90, 95% CI: 0.83–0.98), indicating that the potential presence of publication bias did not affect the overall conclusions. Furthermore, the analyses of clinical signs (*t* = −2.109, *p* = 0.05) and fever (*t* = −1.993, *p* = 0.06) did not yield statistically significant results, suggesting that publication bias was unlikely to have influenced these specific disease outcomes.

## 4 Discussion

ASFV poses a significant threat to the global swine industry because of its severe morbidity and mortality rates. The complex genome structure of the virus, intricate life cycle, and absence of an effective vaccine pose remarkable challenges in controlling this devastating disease. Various vaccine platforms have been explored to address the challenges of vaccine development; each platform has distinct advantages and limitations. Given the safety concerns associated with live-attenuated vaccines, non-replicating vaccines have been explored as safer alternatives for ASFV control. These vaccines are designed without live pathogens capable of replication; instead, they use inactivated or non-viable components of a pathogen to elicit an immune response without causing illness (83). This category also includes viral vector-based vaccines that utilize replication-deficient viral vectors (84, 85). Thus, the current manuscript provides a quantitative evaluation of the efficacy of non-replicating vaccines developed to protect domestic pigs against ASFV infection. The analysis focused on VE by examining three key disease outcomes: mortality, fever, and clinical symptoms after the infection.

The meta-analysis revealed that vaccination with subunit vaccines significantly reduced the mortality risk in vaccinated pigs compared to that in control pigs (*p* = 0.02), with a RR of 0.90 (95% CI: 0.83–0.98). This effect size suggests that 10% of the vaccinated pigs were protected against virulent ASFV. Notably, no significant heterogeneity was detected among the studies (*I^2^* = 16, *p* = 0.26), indicating consistent results across the different trials (Figure 4). Although statistically significant results can be compelling, they should be interpreted with caution when assessing the clinical, epidemiological, and economic relevance of a vaccine. On one hand, even a vaccine with a small effect size could offer substantial value in contexts where partial protection aids in disease control, especially in cases where the disease has a devastating impact. On the other hand, a very high statistically significant effect may not always translate into a meaningful impact if the vaccine does not reduce morbidity, mortality, or transmission rates, especially for diseases with high economic and epidemiological impact like ASF. As illustrated in Figures 4, 6, while the reduction in mortality was statistically significant, the practical value of subunit vaccines remains limited owing to the minimal protection they provide against fever and other ASF-related symptoms. ASFV presents a distinctive challenge owing to its genetic complexity, boasting a sizable genome that encodes approximately 150–167 open reading frames (ORFs), depending on the strain (4, 40). This genetic intricacy limits the selection of antigenic determinants and epitopes capable of eliciting strong, prolonged immunity, thus rendering the development of an effective subunit vaccine extremely complex (27, 31).

In clinical trials, killed vaccines have consistently failed to protect pigs from ASFV, even when exposed to homologous strains (82). Research has indicated that effective protection against ASFV is closely linked to antigen-specific antibodies and CD8 + T-cell responses (6, 31). The poor efficacy of inactivated vaccines is often attributed to their inability to elicit robust humoral and cellular immunity after administration (12, 86). These vaccines cannot replicate or infect host cells, they do not trigger antigen processing via the MHC-I pathway and thus poorly prime CD8^+^ cytotoxic T-cell responses necessary for clearing infected macrophages (1). Moreover, although antibodies are elicited, they are typically non-neutralizing and fail to prevent ASFV replication, and in some cases may even enhance disease severity (26). Furthermore, common inactivation methods (e.g., chemical or irradiation) can impair critical conformational epitopes and reduce antigen integrity, undermining effective antibody binding (87, 88). To enhance the immunogenicity of inactivated vaccines, recent formulations have incorporated advanced adjuvants, such as Polygen and Emulsigen D. These adjuvants are designed to stimulate both humoral and cellular immune responses, including interferon gamma production, which is essential for protection against ASFV (12, 33). Despite these groundbreaking discoveries, the current meta-analysis revealed that vaccination of pigs with inactivated vaccines did not confer any protection against mortality (RR = 1.01, 95% CI: 0.95–1.06), fever (RR = 1.00, 95% CI: 1.00–1.00), or other ASF-related symptoms (RR = 1.00, 95% CI: 1.00–1.00) when compared to that in control groups (Figure 7). These findings clearly indicate that the use of inactivated or killed vaccines is not a feasible strategy and holds limited promise for preventing ASF. According to Pikalo et al. (2022), the effective generation of robust cell-mediated immunity typically requires viral replication within the host (34), which may explain the lower efficacy of inactivated and subunit vaccines in comparison to that of live attenuated vaccines. Furthermore, the complexity of virions, coupled with their intracellular and extracellular localization, makes viral neutralization difficult and often results in the creation of antibodies that provide no protection or potentially worsen the disease (26, 27, 79).

Unlike protein-based subunit vaccines, DNA and vector-based vaccines are more immunogenic (36). This is because they enable intracellular antigen expression, allowing presentation via MHC-I pathways, which is crucial for activating CD8^+^ T cells (4, 36). The recognition of vector-associated pathogen-associated molecular patterns (PAMPs) such as dsRNA or viral proteins by pattern recognition receptors (PRRs), including TLR3, TLR7, TLR9 in endosomes, and the cGAS–STING cytosolic DNA-sensing pathway, initiates dendritic cell (DC) maturation and triggers type I interferon (IFN-*α*/*β*) production (89). This signaling also upregulates co-stimulatory molecules and induces robust secretion of cytokines like IL-12p70, TNF-α, and IFN-*γ*, which collectively bridge innate sensing to adaptive immunity, leading to effective CD4^+^ and CD8^+^ T-cell responses necessary for clearance of infected macrophages (89). However, this contrasts with the findings of the subgroup analysis. The results showed no statistically significant differences in protection between pigs vaccinated with protein-based vaccines, DNA-based vaccines, viral-vectored vaccines, or their combinations (*p* = 0.31), as illustrated in Figure 5. This clearly indicates that the mechanisms underlying ASFV protection remain poorly understood, and the significance of antibody-mediated and cell-mediated immune responses in ASFV protection is yet to be elucidated (47). Antibody-dependent enhancement (ADE) of ASFV infection and disease progression is common, as demonstrated in multiple studies involving swine immunization with attenuated or subunit vaccines (12, 66, 79). Additionally, previous research has highlighted the pivotal role of CD8^+^ T cells in viral clearance, albeit the presence of IFNγ-specific T cells alone does not guarantee complete protection against ASFV infection and disease (47, 48). Further investigations are essential to determine the optimal antibody response that ensures protection while avoiding the harmful effects of excessive antibody production. From an applied perspective, this understanding is crucial for the development of effective and safe immunization strategies.

This study had a few limitations. Although the search strategy effectively retrieved the most relevant studies from electronic databases without language restrictions, the exclusive use of English-spelled search terms may have resulted in the omission of studies published in other languages. The fact that only four studies on inactivated vaccines met our inclusion criteria reflects a limited evidence base, which may reduce analytical robustness and external validity. This scarcity undermines statistical power and constrains our ability to generalize findings across diverse swine populations and real-world settings. Consequently, any conclusions regarding the effectiveness of inactivated vaccines must be considered preliminary and context-specific, rather than definitive. We also acknowledge that we did not plan to contact vaccine companies or research institutes for unpublished data that could have been eligible for inclusion. Moreover, the findings indicated a potential publication bias for the mortality outcomes, which could have affected the statistical power in pooling the effect size. However, the presence of publication bias should be interpreted with caution as it does not necessarily pose a threat to the validity of the findings. In fact, after applying the trim-and-fill method, the adjusted effect size was estimated to be 0.89 (95% CI: 0.80–1.00), which is comparable to the overall pooled effect size of 0.90 (95% CI: 0.83–0.98) as reported previously. Thus, publication bias may exist; however, it does not have a significant impact on the estimated effect size. Additionally, the variations in vaccination protocols, particularly regarding the breed and age of pigs included in the trials, along with small sample size of animals used in the vaccine experiments, pose significant limitations. Consequently, extrapolating experimental findings to natural field settings should be approached with caution to ensure accurate interpretation and applicability. Furthermore, although many trials have evaluated humoral and cellular immune responses, and viral shedding post ASF vaccination or infection, these data were excluded from the meta-analysis. This exclusion may have affected the assessment of immune response dynamics and the related findings available in the scientific literature.

## 5 Conclusion

This study provided a comprehensive assessment of the effectiveness of current non-replicating vaccine candidates in protecting pigs against ASFV. Compared to live-attenuated vaccines, non-replicating vaccines can deliver significant benefits, including enhanced specificity, stability, and safety, while also enabling DIVA. Overall, the findings of this study indicate that the use of inactivated vaccines represents an unsuccessful strategy and holds limited promise for preventing ASF. In contrast, subunit vaccines against ASFV provide approximately 10% protection against mortality and 3% protection against fever and other clinical symptoms in domestic pigs. From a scientific perspective, this marks a significant step forward in the pursuit of an ASF vaccine, suggesting that the development of an effective subunit vaccine is a realistic goal with continued efforts. However, from a practical standpoint, the clinical, epidemiological, and economic relevance of currently available subunit vaccines remains limited. This limitation arises because most vaccinated pigs developed viremia or a chronic form of ASF, which may pose a serious threat to animal health and complicating disease control efforts. The virus’s persistence and potential for undetected spread undermine control measures. At present, subunit vaccines are likely to serve as a long-term choice in vaccine development strategies. Further research is essential to deepen our understanding of the roles and significance of humoral and cellular immune responses against ASFV infection, as well as to identify critical viral antigens and delivery systems that can induce effective protective immunity.

## Data Availability

The original contributions presented in the study are included in the article/Supplementary material, further inquiries can be directed to the corresponding author/s.
